# Non-invasive SpO2/FiO2 ratio (SFR) as surrogate for PaO2/FiO2 ratio (PFR): A scoping review

**DOI:** 10.2478/jccm-2025-0024

**Published:** 2025-07-31

**Authors:** Madhura Reddy, Malavika Kulkarni, Sushma Thimmaiah Kanakalakshmi, Laxmi Shenoy, Rama Rani KrishnaBhat

**Affiliations:** Department of Respiratory Therapy, Manipal College of Health Professions, Manipal, Manipal Academy of Higher Education, Manipal, Karnataka, India; Department of Anesthesiology, Kasturba Medical College, Manipal, Manipal Academy of Higher Education, Manipal, Karnataka, India

**Keywords:** SFR, PFR, oxygen, oxygenation status, non-invasive tool

## Abstract

Patient oxygenation significantly impacts clinical outcomes, and continuous monitoring is essential, especially in critical care settings where hypoxia is the leading cause of mortality. PFR (PaO2/FiO2 ratio or P/F ratio) is an invasive method for measuring oxygenation requiring arterial blood gas (ABG) sampling, however it carries complications making non-invasive methods more desirable. SFR (SpO2/FiO2 ratio or S/F ratio), a non-invasive tool based on pulse oximetry, provides a cost-effective and rapid way to monitor oxygenation status, especially in settings where advanced methods are unavailable. A total of 575 articles were screened from databases including Web of Science, Scopus, PubMed, and CINAHL, with 32 articles meeting the inclusion criteria for this scoping review wherein SFR was used as a surrogate for PFR and a diagnostic tool for acute lung injury and ARDS. A total of 81,637 patient records were analyzed, including ABG values, pulse oximetry readings, mechanical ventilator settings, and patient diagnoses. The study population included adults, pediatric patients, and neonates admitted to critical care units, with common diagnoses including acute hypoxemic respiratory failure, ARDS, and COVID-19. In the context of COVID-19, SFR was used to predict the need for mechanical ventilation, with a cut-off of 300 indicating a threshold for imminent ventilation requirement. The studies demonstrated statistically significant sensitivity and specificity for SFR, highlighting its utility as a non-invasive tool for assessing oxygenation status. SFR has shown potential as a reliable non-invasive surrogate for determining oxygenation status across all populations.

## Introduction

Patient oxygenation significantly impacts overall clinical outcome in critical care settings. A large proportion of critical care admissions are due to acute respiratory distress syndrome (ARDS), making continuous monitoring of oxygenation essential. Hypoxia remains the leading cause of mortality among ICU (intensive care unit) patients, emphasizing the importance of timely detection and prevention. Clinicians employ various techniques to monitor oxygenation in critically ill patients, which can be invasive or non-invasive [1,2,3]. The most common invasive method for assessing oxygenation status in adults involves calculating the ratio of partial pressure of arterial oxygen (P_a_O_2_) to the fraction of inspired oxygen (FiO_2_) (also known as PFR). The PFR is being utilized widely to determine the oxygenation status and severity of lung disease and oxygen index predicts both mortality and the duration of invasive ventilation [1,2]. However, in the neonatal population, the oxygen index (OI=mean airway pressure × FiO_2_ × 100 ÷PaO_2_) is a critical parameter to assess severity of hypoxic respiratory failure, but both methods rely on values obtained through arterial blood gas (ABG) sampling.

Invasive methods of measuring oxygenation are linked to various complications, including arterial puncture, a higher risk of infection, iatrogenic anemia, excessive blood withdrawal, arterial line blockages, variations in values due to delays between arterial blood gas sampling and analysis, and increased medical costs [4,5,6]. To avoid these complications, non-invasive methods for measuring oxygen saturation can be employed. Pulse oximetry is the most basic non-invasive technique for monitoring oxygenation in ICU settings, thus non-invasive index (SpO_2_/FiO_2_; also known as SFR) can be used as a surrogate to invasive PFR. In healthcare facilities where advanced methods for ABG analysis are unavailable or trained personnel to perform the procedure are lacking, reliance on ABG measurements can delay treatment. Pulse oximetry-based non-invasive SFR oxygenation indices provide a more cost-effective and faster way to evaluate oxygenation status while aiding in the early identification of at-risk patients [6,7].

This review paper aims to address the following questions:

To evaluate the extent to which the SFR serves as a reliable non-invasive surrogate for the PFR in determining oxygenation status.
To explore whether the SFR can be applied across adult, pediatric, and neonatal populations for diagnosing disease conditions or as a predictive tool for assessing treatment response.

## Methods

### Study search:

The study protocol was drafted concerning the Preferred Reporting Items for Systematic Reviews and Meta-Analyses extension for Scoping Reviews (PRISMAScR) [8]. We did a comprehensive search for articles by accessing databases such as Scopus, PubMed, Web of Science, and CINAHL database. Keywords used to find articles included SFR, PFR, oxygenation status, oxygen, and non-invasive tool. Articles from 2000 to 2024 were searched and Rayyan.ai software was used to delete any duplicates.

### Inclusion criteria

**Population**: Adult, pediatric, and neonates

**Intervention**: Comparison of SFR with PFR in determining the utility of non-invasive tools. Use of SFR in combination with other parameters to determine the success or failure of any critical care treatment and in diagnosing any pulmonary conditions. Title and abstract screening, followed by full-text screening, was conducted by two reviewers using a standardized template aligned with the inclusion and exclusion criteria.

## Results

### Study selection

**Fig. 1.**
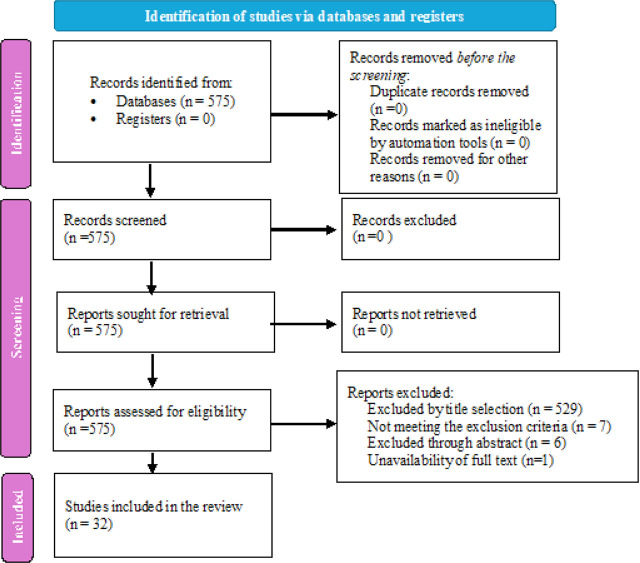
PRISMA flow diagram

A total of 575 articles were initially screened, focusing on the title, abstract, and availability of full text for this scoping review. The search databases used were Web of Science (433), Scopus (112), PubMed (29), and CINAHL (1). Data extracted from the articles included the year of publication, study location, population size, diagnosis, use of the SFR, and outcomes. After applying the inclusion and exclusion criteria, 32 articles were included in the final review. Articles excluded during title selection primarily focused on medication effects on critical care diseases, therapeutic interventions for critical care disorders, or other parameters impacting oxygenation that did not specifically examine the SFR or PFR. After screening titles, abstracts were reviewed, leading to further exclusions of studies that did not align with our inclusion criteria. Only one article, initially deemed relevant based on title and abstract, was excluded due to the unavailability of the full text. The article screening process is outlined in detail in Figure 1.

### Study characteristics

Most studies included in the review were published between 2020 and 2024. Geographically, most studies were conducted in the United States of America, India, South Korea, Spain, Thailand, France, Italy, Japan, Pakistan, Brazil, China, and Iran. The majority of the studies were retrospective in design [9], with a few prospective observational studies and two cross-sectional studies. No randomized controlled trials were included. To statistically evaluate the reliability of the SFR, univariate and multivariable logistic regression models were used, along with receiver operating characteristic (ROC) analysis and area under the curve (AUC) to assess the performance of this non-invasive tool. Regression analysis was also employed to establish the relationship between the SFR and PFR. The studies included are detailed in Table 1.

**Table 1.** Characteristics of the studies

**Title of the study**	**Authors**	**Sample size**	**Inclusion criteria**	**Year/Place**	**Methodology**	**Conclusion**
Comparison of the SpO2/FIO2 ratio and the PaO2/FIO2 ratio in patients with acute lung injury or ARDS	Rice et al.	672	ARDS ventilated with low tidal volume strategy	2007/USA	Corresponding measurements of Spo2 (values ≤ 97%) and Pao2 from patients enrolled in the ARDS Network trial of a lower tidal volume ventilator strategy (n = 672) were compared to determine the relationship between SFR and PFR. SFR threshold values correlating with PFR ratios of 200 (ARDS) and 300 (ALI) were determined.	SFR of 235 and 315 correlated with PFR of 200 and 300, respectively, for diagnosing and following up patients with ALI and ARDS.
Comparison of the Pulse Oximetric Saturation/Fraction of Inspired Oxygen Ratio and the Pao2/Fraction of Inspired Oxygen Ratio in Children	Khemani et al.	1298	Children with acute lung injury	2009/Los Angeles	Electronically queried blood gas measurements from two tertiary care pediatric ICUs (PICUs). Included in the analysis were corresponding measurements of Spo2, Pao2, and Fio2 charted within 15 min of each other when Spo2 values were between 80% and 97%.	SFR is a reliable noninvasive marker for PFR to identify children with ALI or ARDS.
Pulse oximetry saturation to fraction inspired oxygen ratio as a measure of hypoxia under general anesthesia and the influence of positive end-expiratory pressure	Tripathi et al.	2754	Surgical patients apart from cardaic and thoracic surgery	2010/USA	Adult general anesthetics performed with arterial blood gas analysis. Intraoperative data were collected from an anesthesia information system. The SFR corresponding to PFR of 300 were determined.	SFR correlated with the PFR in patients undergoing general anesthesia, especially those ventilated with PEEP more than 9 cm H2O and/or with P/F less than 300.
Oxygen saturation/fraction of inspired oxygen ratio is a simple predictor of noninvasive positive pressure ventilation failure in critically ill patients	Spada C et al.	133	Respiratory failure patients receiving NIV support	2010/USA	A prospective observational study was conducted in patients requiring NPPV. Clinical data including respiratory mechanics at the time of NPPV initiation, and clinical outcomes were recorded.	A simple index of SFR at the time of NPPV initiation could be used to identify patients at high risk of NPPV failure
The use of the pulse oximetric saturation/fraction of inspired oxygen ratio for risk stratification of patients with severe sepsis and septic shock	Serpa Neto et al.	260	Patients with severe sepsis	2013/Brazil	Retrospective cohort study in those admitted to 2 tertiary mixed intensive care units with severe sepsis or septic shock.	Low SFR upon ICU admission is associated with an increased risk of mortality in patients presenting with septic shock.
SpO2/FiO2 as a predictor of non-invasive ventilation failure in children with hypoxemic respiratory insufficiency	Pons-Odena et al.	309	Children with acute respiratory failure	2013/Spain	Data of children requiring NIV support were included. Clinical data collected were RR, heart rate, SpO2 and FiO2 before NIV was started.	SFR is a reliable predictor of early NIV failure in children.
SpO2/FiO2 Ratio on Hospital Admission Is an Indicator of Early Acute Respiratory Distress Syndrome Development Among Patients at Risk	Festic et al.	4646	Pateints admitted in acute care setting with atleast one risk factor for ARDS.	2013/USA	SFR was noted in the first 6 hours of admission. SFR was evaluated as a continuous as well as a categorical variable by creating the following arbitrarily chosen SFR cutoffs: <100, 100 < 200, and 200 < 300. These simplified cutoffs were chosen empirically to approximate the PFR cutoffs for ARDS severity	SFR measured within first 6 hours of admission to the hospital is an independent indicator of early ARDS development among patients at risk. Lesser SFR may indicate earlier progression to fully established ARDS.
Correlation of oxygen saturation as measured by pulse oximetry/fraction of inspired oxygen ratio with PaO2/fraction of inspired oxygen ratio in a heterogeneous sample of critically ill children	Lobete et al.	298	Children under mechanical ventilation, noninvasive ventilation support, and breathing spontaneously admitted to a tertiary care noncardiac surgery PICU.	2013/spain	A retrospective database study was conducted in a pediatric intensive care unit of a university hospital. Simultaneous blood gas and pulse oximetry were collected in a database.	Oxygen saturation as measured by SFR is an adequate noninvasive surrogate marker for PFR.
The use of the pulse oximetric saturation to fraction of inspired oxygen ratio in an automated acute respiratory distress syndrome screening tool	Schmidt et al.	3767	All patients admitted in critical care unit	2015/USA	This was a retrospective cohort study using the Multiparameter Intelligent Monitoring in Intensive Care II database. The relationship was derived and validated in all patients ventilated for at least 24 hours.	The SFR may be an adequate substitute for the PF ratio in an automated ARDS screening tool.
Correlation of SpO2/FiO2 Ratio and PaO2/FiO2 Ratio in Hypoxemic Patient While Breathing in Room Air.	Nittha Oerareemitr et al.	179	Hypoxemic patients on room air	2018/Thailand	Spo2 and pao2 measurements were done on room air.	SFR from pulse oximetry significantly correlated with PFR from ABG analysis but the exact equation was not as good enough to estimate PFR calculated from the SFR of the pulse oximetry.
Spo2/fio2 on presentation as a predictor for early hemodynamic deterioration in intermediate risk acute pulmonary embolism	Domaradzki et al	178	Intermediate-risk pulmonary embolism (hemodynamically stable with right ventricle to left ventricle ratio > 0.9 or tricuspid annular plane systolic excursion < 18 mm).	2019/USA	For patients admitted through the emergency department. the first available set of parameters was taken into account; For in-patients, the parameters were recorded at the closest time to diagnostic suspicion and before any intervention. SFR on presentation was computed by using a conversion table as follows	In intermediate-risk pulmonary embolism, SFR on presentation can help predict the risk of early hemodynamic deterioration
Continuously available ratio of SpO2/FiO2 serves as a noninvasive prognostic marker for intensive care patients with COVID-19	Lu Xiaofan et al.	280	Severe and critically ill (intensive care) patients with COVID-19.	2020/China	The ratio of SFR was measured at day 1, 3, 7, 14 and 28 since admission to intensive care wards.	SFR could serve as a non-invasive prognostic marker to facilitate early adjustment for treatment, thus improving overall survival.
Pulse oximetric saturation to fraction of inspired oxygen (SpO2/FIO2) ratio 24 hours after high-flow nasal cannula (HFNC) initiation is a good predictor of HFNC therapy in patients with acute exacerbation of interstitial lung disease	Koyauchi T et al.	66	Acute exacerbation of interstitial lung disease	2020/Japan	Retrospective analysis of patients with AE-ILD who underwent HFNC. Overall survival, the success rate of HFNC treatment, adverse events, temporary interruption of treatment, discontinuation of treatment at the patient's request, and predictors of the outcome of HFNC treatment were evaluated.	The SFR 24 hours after initiating HFNC was a good predictor of successful HFNC treatment.
SpO2/FiO2 as a predictor of high flow nasal cannula outcomes in children with acute hypoxemic respiratory failure	Kim Ga Eun et al.	139	Children treated with HFNC due to AHRF	2021/South korea	Trends of SFR and PFR during HFNC were analyzed. To predict HFNC outcomes, a nomogram was constructed based on predictive factors.	SFR may be an easy-to-use predictor of HFNC outcomes in children with AHRF
Mortality Prediction Using SaO2/FiO2 Ratio Based on eICU Database Analysis	Patel Sharad et al.	33701	18 years and above patient requring all modalities of oxygen supplementation	2021/USA	The features age, gender, SaO2, PaO2, FIO2, admission diagnosis, Apache IV, mechanical ventilation, and ICU mortality were extracted from the eICU Collaborative Research Databas	SFR appears to be a better predictor of ICU mortality than PFR.
Role of SpO2/FiO2 Ratio and ROX Index in Predicting Early Invasive Mechanical Ventilation in COVID-19. A Pragmatic, Retrospective, Multi-Center Study	AlberdiIglesias et al.	2040	Participants with suspected COVID-19 infection and those transferred with high priority by ambulance to the corresponding ED	2021/Spain	Multicenter, retrospective cohort study was	SFR had better accuracy than the ROX index in predicting Invasive MV. SFR is a simple, non-invasive, and promising tool for predicting the risk of IMV in patients infected with COVID-19.
Assessment of the SpO2/FiO2 ratio as a tool for hypoxemia screening in the emergency department	Catoire P et al	395	COVID-19 pateints	2021/France	Retrospectively studied patients admitted to an academic-level ED who were undergoing a joint measurement of SpO2 and arterial blood gas. Compared SpO2 with SaO2 and evaluated performance of the SFR for the prediction of 300 and 400 mmHg PFR cut-off values	SFR showed a good association with the PFR. SFR can be used for the estimation of the degree of hypoxemia on admission to the emergency room, allowing the patient's severity to be assessed prior to confirmation of viral status.
Correlation of Pao2/Fio2 Ratio with Spo2/Fio2 Ratio in Children on Mechanical Ventilation	Zahra ahmed et al.	30	Children requring atleast 48 hours of mechanical ventilation in PICU	2021/Pakistan	Arterial blood gas sampling for calculation of PFR and measurement of SFR was done simultaneously (within 5 minutes).	Noninvasive SFR can reliably be used in place of PFR in children on mechanical ventilation as a strong correlation was observed
Correlation Between the Ratio of Oxygen Saturation to Fraction of Inspired Oxygen and the Ratio of Partial Pressure of Oxygen to Fraction of Inspired Oxygen in Detection and Risk Stratification of Pediatric Acute Respiratory Distress Syndrome	Lohano PD et al.	120	Age range of 2 months to 16 years, admitted to PICU with acute onset of respiratory distress	2021/Pakistan	Measured SpO2, PaO2, FiO2 and calculated SFR and PFR	Strong correlation between the SFR and PFR, and a statistically substantial agreement. So, the SFR can be reliably used for early detection and risk assessment of ARDS in children.
S/F and ROX indices in predicting failure of high-flow nasal cannula in children	Kim Ji Hye et al	152	Children with respiratory distress	2022/South korea	Ratio of percutaneous oxygen saturation to the fraction of inspired oxygen (S/F), the ratio of SFR to RR (ROX), the ratio of SFR to RR/median RR (ROX-M), and the ratio of SFR to z-score of RR (ROX-Z) were calculated and compared between groups	SFR and ROX-M can be used for early prediction of hypoxic HFNC failure.
Correlation of SpO(2)/FiO(2) and PaO(2)/FiO(2) in patients with symptomatic COVID-19: An observational, retrospective study	Bonaventura Aldo et al.	1028	>18 years, symptomtic COVID 19 patients; patients with >97% SpO2 were excluded.	2022/Italy	To ensure accuracy of SpO2 assessments, the following considerations were followed: (1) no position changes 5 min prior to the measurement; (2) checking for the accurate position and cleanliness of the sensor; and (3) evaluation of satisfactory waveforms on the monitor. After 1 min of steady SpO2 measurement, the value was recorded along with the oxygen setting,	Routine use of SFR as a reliable surrogate of PFR in patients with COVID-19-related ARDS.
Utility of Pulse Oximetry Oxygen Saturation (SpO2) with Incorporation of Positive End-Expiratory Pressure (SpO2*10/FiO2*PEEP) for Classification and Prognostication of Patients with Acute Respiratory Distress Syndrome	Todur Pratibha et al.	85	Patients aged 18–80 years on invasive mechanical ventilation (MV) diagnosed with ARDS	2022/India	The values of PaO2, FiO2, and SpO2 were collected at three different time points. They were at baseline, i.e., after intubation and initiation of MV (within one hour of intubation), day one (1–24 hours of MV), and day three (48–72 hours of MV).	S/FP *10 has a strong correlation to P/FP *10 in ARDS patients.
ROX index and SpO2/FiO2 ratio for predicting high-flow nasal cannula failure in hypoxemic COVID-19 patients: A multicenter retrospective study	Kim Jin Hyoung et al.	133	COVID-19 pateints receiving HFNC	2022/South korea	The ROX index and the SpO2/FiO2 ratio at 1 h, 4 h, and 12 h after HFNC initiation were calculated	SFR following HFNC initiation was an acceptable predictor of HFNC failure. SFR may be a good prognostic marker for intubation in COVID-19 patients receiving HFNC.
Combining blood glucose and SpO2/FiO2 ratio facilitates prediction of imminent ventilatory needs in emergency room COVID-19 patients	Sakai K et al.	106	COVID 19 patients requring oxygen support	2023/Japan	Blood glucose and SFR was recorded as they were easliy available.	Measuring blood glucose and SFR may be a simple and versatile new strategy to accurately identify ER patients with COVID-19 at high risk for the imminent need of MV.
Analysis of ROX Index, ROX-HR Index, and SpO2/FIO2 Ratio in Patients Who Received HighFlow Nasal Cannula Oxygen Therapy in Pediatric Intensive Care Unit	Choi Sun Hee et al	107	Children admitted to PICU	2023/South korea	Data on clinical and personal information, ROX index, ROX-HR index, and SFR were collected	ROX index, ROX-HR index, and SFR appear to be promising tools for the early prediction of treatment success or failure in patients initiated on HFNC for acute hypoxemic respiratory failure.
Ratio of Oxygen Saturation to Inspired Oxygen, ROX Index, Modified ROX Index to Predict High Flow Cannula Success in COVID-19 Patients: Multicenter Validation Study	Ruangsomboon Onlak et al.	173	Adult patients with COVID-19 treated with HFNC in the ED	2023/Thailand	All these parameters were measured while the patients were still in the ED awaiting disposition. At 0 and 2 hours after HFNC initiation, we calculated three parameters and assessed them for their utility in predicting HFNC outcomes: the SFR; the ROX index; and the modified ROX index.	The SFR measured two hours after high-flow nasal cannula initiation was better than the ROX index and the modified ROX index at predicting HFNC success in patients with acute hypoxemic respiratory failure secondary to COVID-19 in the ED setting.
Comparison of PaO2/FiO2 (PF ratio) to SpO2/FiO2 (SF ratio) and OI to OSI for Predicting Short Term Outcomes in Children with Acute Hypoxemic Respiratory Distress: A Prospective Observational Study	Shah Niyati et al.	200	Children with acute hypoxemic respiratory distress	2024/India	Prospective observational study Serial PFR and SFR calculated at 0, 6, 24 and 48 hours were compared and their trends were utilized for prediction of 28 day mortality. Same was done in ventilated patients using OI and OSI.	SFR is a reliable surrogate for PFR and a useful predictor of progression to ventiation and survival at discharge
Emergency Department SpO2/FiO2 Ratios Correlate with Mechanical Ventilation and Intensive Care Unit Requirements in COVID-19 Patients	Zhang Gary et al.	539	Adult COVID-19 patients	2024/USA	Retrospective chart review Highest and lowest SFR were calculated on admission	SFR of 300 or below correlated with the need for mechanical ventilation during hospitalization.
SpO2/FiO2 and PaO2/FiO2 for Predicting Intensive Care Admission in Wheezy Children: An Observational Study	Beniwal Rakhi et al.	315	Wheezy children aged 6 months to 12 years requiring admission in the pediatric emergency department	2024/India	Oxygen saturation (SpO2) and fraction of oxygen in inspired air (FiO2) were recorded at admission while the partial pressure of oxygen (PaO2) was measured using arterial blood gas analysis performed within half an hour of admission	SFR cut-off of < 300 had a good sensitivity in determining need for PICU admission. SFR had only a moderate correlation with PFR.
A Prospective Observational Study Comparing Oxygen Saturation/Fraction of Inspired Oxygen Ratio with Partial Pressure of Oxygen in Arterial Blood/Fraction of Inspired oxygen ratio among critically Ill patients requiring different modes of Oxygen supplementation in ICU	Alur Rakesh et al	125	Adult AHRF (acute hypoxemic respiratory failure) patients receiving oxygen therapy	2024/India	At admission and during deterioration or within 24 hours, measurements of FiO2, PaO2, and SpO2 were documented	Uutility of the SFR as a substitute for the PFR in the diagnosis of AHRF in adults who are critically ill.
Evaluation of Correlation and Agreement between SpO2/FiO2 ratio and PaO2/FiO2 ratio in Neonates	Muniraman H et al	196	Neonates with respiratory failure	2022/USA	Retrospective cohort study including neonates with respiratory failure over a 6-year study period. Correlation and agreement between PFR with SFR was analyzed	SFR correlated strongly with PFR with good agreement between derived PFR from noninvasive SpO2 source and measure PFR.
Comparison of non-invasive to invasive oxygenation ratios for diagnosing acute respiratory distress syndrome following coronary artery bypass graft surgery: a prospective derivation-validation cohort study	Bashar Farshid R et al.	671	Pateints undergoing CABG surgery	2018/Iran	SPO2, PaO2, and FiO2 were measured once per patient upon study enrollment, and SPO2 was recorded at the time of ABG sampling. SPO2 was observed for a minimum of 1 min before the value was recorded.	PaO2 and SaO2 correlated in the diagnosis of ARDS, with a PFR of 300 correlating to an S/F of 311 (Sensitivity 90%, Specificity 80%). The SFR may allow for early real-time rapid identification of ARDS.

### Study population

A total of 81,637 patient records were collected, including arterial blood gas values, primarily the PFR, vital signs (mainly pulse oximetry values), mechanical ventilator settings such as F_i_O_2_, and patient diagnoses. The study population included adults, pediatrics, and neonates admitted to critical care units for various respiratory illnesses. The primary focus was on the intensive care setting, where most respiratory-related diseases are observed. The most common pathologies/diagnoses among these patients were acute hypoxemic respiratory failure, acute lung injury, and acute respiratory distress syndrome (ARDS)[10,11,12,13,14,15,16,17,18,19,20,21,22] followed by COVID-19 (SARS-CoV-2) [23,24,25,26,27,28,29,30], cardiothoracic surgeries [31,32], sepsis [33], pulmonary embolism [34], asthma [35], and interstitial lung disease [36]. Some studies included data from all patients admitted to critical care during a specified period, covering all age groups [37,38,39,40,41].

### SFR as a surrogate tool

Almost all the studies included in this review have utilized SFR in some capacity to assess its utility in daily clinical practice. Many studies have evaluated SFR as a surrogate for PFR, while others have examined its effectiveness as a diagnostic tool, particularly for diagnosing acute lung injury and ARDS in both adult and neonatal populations. Some studies have investigated SFR as a predictor for success or failure in high-flow nasal cannula (HFNC) therapy. A few studies have explored the use of SFR in combination with other tools, such as blood glucose [25], ROX index [8,24, 26,29,41] and oxygenation saturation index (OSI) [17,19,21,24,37] to predict weaning outcomes. One study focused on using SFR to predict mortality in critically ill patients [39]. Additionally, some studies evaluated the SFR role in determining hemodynamic deterioration in pulmonary embolism [34]. Few studies assessed whether SFR could serve as a stand-alone tool for diagnosing ARDS [10,14,21,28,29], the success of High flow nasal cannula (HFNC) therapy [24,26] and for screening hypoxia [28,31,37]. In essence, all these studies aimed to replace PFR with SFR to assess the efficacy of the latter as a non-invasive alternative and found that SFR can be replaced and used to determine the various outcomes in critical care set-up. The use of SFR was not only limited to critical care settings but was also employed in emergency departments [23,28] to quickly assess the need for oxygen therapy and identify the worsening of lung conditions leading to ARDS or the potential need for mechanical ventilation. In the COVID-19 population, SFR was primarily used to monitor the response to therapy, evaluate whether the patient’s condition was improving or deteriorating, and predict the potential need for higher levels of ventilation.

### Optimal Cut-off Value for SFR in clinical practice

The statistical test used to assess the correlation between SFR and PFR was Spearman’s correlation in most of the studies. The area under the curve (AUC) for sensitivity and specificity was employed to evaluate the performance of both SFR and PFR. With a 95% confidence interval (CI), all the studies included in this review demonstrated statistically significant sensitivity and specificity (p value <0.05) for SFR, highlighting the utility of this non-invasive tool.

Various cut-off values were identified for different levels of PFR. In addition, cut-off values were assessed for other purposes, such as predicting ICU admission and determining the success or failure of HFNC therapy. The majority of studies found that an SFR of <300 indicated worsening of respiratory condition or hypoxemia and a need for mechanical ventilation. However, the various cutoff values identified were; for a PFR of 300, the identified cut-off values for SFR were 241(AUC 0.74; 95% CI) [33], 315 (Positive likelihood ratio 2.06, 95% CI of 1.64 – 2.76) [10], 263 [for ALI – 93% sensitivity; 95% CI (264 – 272)] [11], 201 201 [for ARDS – 84% sensitivity, 95% CI 206 – 211)] [11], 296 [95% CI (292–314)] [37], 321 (AUC 0.87; sensitivity −68.9%, specificity – 95%) [21], and 311 (Sensitivity 90%, Specificity 80%) [32]. Another study found that a cutoff of SFR <433 (AUC 0.95; p < 0.001) accurately identified ARDS (as defined by PFR of <300) [27]. These values varied depending on the disease condition and the time of data collection. A threshold of 350 indicates PFR < 300 [AUC: 0.91 (CI 95% 0.885–0.950)], while a threshold of 470 indicates PFR < 400 [AUC:0.9 (95% CI 0.872–0.930)] among COVID-19 population [28]. One study found that an SFR value of 236 [95% CI (230–245)] [37], 252 (AUC 0.83; sensitivity −68.9%, specificity – 95%) [21] and 235 (positive likelihood ratio 5.64; 95% CI of 4.69 – 7.08) [10] corresponds to a PFR of 200 or less, while an SFR value of 146 [95% CI (140–148)] corresponds to a PFR of 100 or less [37]. To identify moderate ARDS (identified by PFR <200), SFR of <336 was found to be moderately accurate (AUC 0.71, 95% CI 0.944–0.969, p < 0.001) [27]. One study found an SFR of 154 (AUC 0.92, 95% CI) [33] corresponded to a PFR of 100. No single cut-off value was established for a specific PFR.

Several studies in the review identified cut-off values for different clinical purposes. One study found that an SFR of 230 predicted failure of high-flow nasal cannula (HFNC) therapy, with a sensitivity of 78.0% and specificity of 68.7% (95% CI 5.06–35.84) [16]. The SFR was also used to predict ICU admission in pediatric patients, with a cut-off value of <300 for predicting PICU admission in wheezy children, and a best predictive cut-off of <178.79 [AUC (95% CI) 0.841 (0.767 – 0.914)] for determining the need for intensive care [35]. In the context of COVID-19 in adults, one study suggested a SFR cut-off of 300, which indicated a threshold for predicting the imminent need for mechanical ventilation^25^. Additionally, a study using the SFR to predict mortality found that mortality significantly increased when the SFR dropped below 200, identifying this as a critical cut-off for assessing hypoxemia [39]. A cutoff value of <98.5 (AUC 0.68, p <0.05) predicted non-invasive positive pressure ventilation in one of the studies [12]. Another finding suggests that a cutoff value of <260 (AUC 0.81; p < 0.05) predicts hemodynamic deterioration, demonstrating a sensitivity of 74% and a specificity of 88% [34].

## Discussion

This review represents the first comprehensive exploration of SFR as a non-invasive surrogate for PFR in determining oxygenation status and its associated clinical outcomes. The aim of this review was to evaluate the utility of SFR across various patient populations and clinical settings. Our findings suggest that SFR can be a reliable tool for assessing oxygenation and patient response in the clinical environment. All studies included in this review reported positive outcomes, highlighting the potential of SFR as a valuable non-invasive measurement. The diverse applications of the SFR, ranging from predicting the need for mechanical ventilation to monitoring treatment response, underscore its clinical relevance.

To summarize, our search included articles from the year 2000 to the present. While the concept of SFR existed earlier, it gained significant attention only in the 2020s. This surge in interest can likely be attributed to the global COVID-19 pandemic, which created an urgent need for a non-invasive tool to assess oxygenation status, particularly as the management of COVID-19 was still evolving. The need for a reliable, non-invasive measure was critical for managing patients and predicting outcomes. In support of this, several studies conducted on COVID-19 patients highlighted SFR as a strong surrogate for predicting the need for mechanical ventilation and ICU admission, as well as assessing the failure of non-invasive mechanical ventilation [12,25]. Beyond COVID-19, SFR was also applied in diagnosing and managing other respiratory conditions, such as asthma in children and pulmonary embolism, demonstrating its broader clinical utility.

Although individual articles had limitations, such as being single-centered and focused on specific regions, our extensive review incorporated data from diverse geographical locations, spanning Europe, Southeast Asia, and South America. Studies conducted across these various ethnic groups indicated that while geographical location might influence the main outcomes of individual studies, it did not significantly impact the findings regarding the use of SFR. This broad geographic representation allows us to confidently generalize the use of SFR as a reliable non-invasive surrogate for PFR, supporting its potential application across different clinical settings and populations.

The majority of the studies included in this review employed a retrospective study design. While retrospective studies have certain limitations, such as potential bias and missing data, they offer advantages in specific contexts. In situations where cut-off values for disease conditions are not yet standardized, retrospective studies provide access to existing data, which can serve as a foundation for planning more robust prospective studies. Although only a few prospective studies have been conducted to date, there is a clear need for additional research to further validate the utility of SFR as a reliable non-invasive tool for assessing oxygenation [9].

Since our review included studies from various countries, its findings can be broadly generalized. Although some individual papers noted that geographical location might influence specific outcomes, these outcomes were not directly related to SFR. Importantly, despite differences in geographical conditions, all studies consistently concluded that SFR is a reliable non-invasive tool for assessing oxygenation status. Moreover, this review encompassed not only the adults but also pediatric and neonatal populations, making the findings applicable across all age groups. SFR has demonstrated excellent sensitivity and specificity, further supporting its utility in diverse clinical settings.

### Growing importance of the SFR in clinical settings

Our review highlights SFR as a valuable alternative tool for assessing oxygenation status, supported by the findings of multiple studies. SFR offers several advantages; first, as a non-invasive method, it avoids complications associated with arterial punctures, such as infection, hemorrhage, and increased costs. Second, it allows for quick acquisition of values, enabling timely evaluation of a patient’s response to therapy and overall oxygenation status.

SFR has demonstrated utility in various clinical scenarios, aiding physicians in decisions such as patient admission to triage, predicting the success or failure of mechanical ventilation strategies, and serving as a diagnostic and screening tool in ARDS settings. While different studies proposed varying cut-off values, a collective cut-off of SFR <300 was commonly used to predict worsening conditions and the potential need for higher levels of mechanical ventilatory support. The application of the SFR across these scenarios underscores its benefits as a practical and effective tool in clinical practice.

Several factors can influence the accuracy and reliability of the SFR in assessing oxygenation status. The PFR provides a more precise measure of oxygenation as it is derived from direct blood gas analysis. However, various clinical factors, such as patient agitation, endotracheal suctioning, and positioning, can impact P_a_O_2_ measurements [33]. Similarly, pulse oximetry readings used to calculate the SFR can be affected by skin pigmentation, nail polish, shock, anemia, and conditions like methemoglobinemia [33, 39]. While these factors may not cause significant variations in cut-off values for predicting deterioration, they should be considered when interpreting results. Additionally, in diseases that severely impair respiratory function, a high prevalence of oxygenation dysfunction can enhance the positive predictive value in statistical analyses. Risk factors contributing to respiratory conditions may further influence the accuracy of the SFR. Therefore, it is crucial to account for these variables before making clinical decisions based solely on SFR measurements [38].

In patients with an S_p_O_2_ of >97%, the values lie on the flat portion of the oxygen dissociation curve. While an S_p_O_2_ >97% is generally indicative of good oxygenation, there are instances where the P_a_O_2_ value may be lower than expected. Since pulse oximetry has an upper saturation limit of 100%, this can influence SFR readings, potentially masking underlying hypoxemia. However, this does not imply that the SFR is an unreliable tool; rather, it underscores the importance of considering other clinical factors and disease conditions when interpreting results to ensure accurate assessment and clinical decision-making [37, 38].

Certain studies have utilized heterogeneous samples, leading to the generalization that the SFR is a reliable tool for assessing oxygenation status, particularly in the pediatric population, regardless of mechanical ventilation status. However, its reliability may be compromised in specific conditions, such as carbon monoxide poisoning, where pulse oximetry readings can be misleading due to the presence of carboxyhemoglobin [37].

Further research is needed to establish a standardized SFR cut-off value comparable to the PFR for practical implementation in assessing oxygenation status. Prospective observational studies and clinical trials focusing on its use in early ARDS detection could help achieve this goal.

A few limitations were identified during this review. A significant number of included studies were retrospective, which inherently limits control over confounding variables. While a few prospective observational studies were reviewed and included to strengthen our findings, their number was relatively small. Additionally, not all studies exclusively focus on assessing the correlation between SFR and PFR. Many incorporated SFR in combination with other indices, such as ROX index, or utilized it as a substitute for PFR, leading to variability in findings. Moreover, while substantial evidence supports the use of SFR in adult populations, there remains a limited number of studies addressing its application in pediatric and neonatal populations. This lack of uniformity and limited data for younger age groups highlight the need for further research to establish more consistent and comprehensive findings.

## Conclusion

SFR has shown potential as a reliable non-invasive surrogate for determining oxygenation status across all populations. Additionally, our findings reveal that SFR can serve as a standalone tool for predicting the severity of ARDS in adult populations. However, to further validate its stability and reliability, more prospective observational studies are necessary in the future. These studies will help establish standardized cut-off values and enhance their applicability across diverse clinical settings and populations.
